# Association of *NOS3* gene polymorphisms with essential hypertension in Sudanese patients: a case control study

**DOI:** 10.1186/s12881-017-0491-7

**Published:** 2017-11-13

**Authors:** Sahar Gamil, Jeanette Erdmann, Ihab B. Abdalrahman, Abdelrahim O. Mohamed

**Affiliations:** 1 Department of Biochemistry, Faculty of Medicine, University of Khartoum, P.O. Box: 102, Khartoum, Sudan; 2 Institute for Cardiogenetics, University of Lübeck, 23562 Lübeck, Germany; 3 DZHK (German Research Centre for Cardiovascular Research), Partner Site Hamburg/Lübeck/Kiel, 23562 Lübeck, Germany; 4 University Heart Center Luebeck, 23562 Lübeck, Germany; 5 Department of Medicine, Faculty of Medicine, University of Khartoum, P.O. Box: 102, Khartoum, Sudan; 6 Neelain Institute for Medical Research, Al-Neelain University, Khartoum, Sudan

**Keywords:** *NOS3*, Essential hypertension, Association, Candidate gene

## Abstract

**Background:**

Essential hypertension (EH) is influenced by various environmental and genetic factors. Nitric oxide is important for the functional integrity of the vascular endothelium and is produced in endothelial cells by the enzyme endothelial nitric oxide synthase (eNOS). EH has a strong genetic component, and the *NOS3* gene, which encodes eNOS, represents an interesting candidate for contribution to the phenotype. The most clinically relevant polymorphisms in the *NOS3* gene are rs1799983 in exon 7 (encoding Glu298Asp), a variable number tandem repeat (VNTR) in intron 4, and rs2070744 (T-786C) in the promoter region. This study aims to investigate the association between these three polymorphisms in the *NOS3* gene and EH in Sudanese patients.

**Methods:**

Hypertensive patients (*n* = 157) > 18 years of age with established hypertension from various hospitals in Khartoum, and controls (*n* = 85) > 18 years of age and with blood pressure measurements <140/90, were included in this case control study. Genotypes at the *NOS3* variants were determined using TaqMan and polymerase chain reaction restriction fragment length polymorphism (PCR-RFLP) analyses. Genotype and allele frequencies were compared between the two groups by χ^2^ analysis, and differences were expressed as odds ratios with 95% confidence intervals (CIs). *P* values <0.05 were considered statistically significant.

**Results:**

The rs2070744 polymorphism in *NOS3* was found to be associated with EH in the Sudanese population as the patients group had higher frequency of CC genotype compared with the controls (6.6% vs 6.1%, *p* = 0.02). Considering a dominant inheritance model, the frequency of TC + CC genotypes in patients was significantly higher than that in the control subjects (52.6% vs 34.1%, respectively; *p* < 0.01), with an odds ratio (95% CI) of 2.14 (1.23–3.74). In addition, the C allele was more frequent in the patients than the control group (29.6% vs 20%, *p* = 0.03, OR = 1.84 (1.15–2.93)). The c allele of intron 4 VNTR was reported in >1% of the Sudanese population under study.

**Conclusion:**

The results of this study indicated that the rs2070744 polymorphism in *NOS3* may be a genetic susceptibility factor for EH in the Sudanese population. The c allele of intron 4 VNTR is not rare in the Sudanese population.

**Electronic supplementary material:**

The online version of this article (10.1186/s12881-017-0491-7) contains supplementary material, which is available to authorized users.

## Background

Essential hypertension (EH) is a multifactorial disease caused by various environmental and genetic factors. The estimated genetic contribution to EH ranges from 25% to 60%, and high blood pressure occurring before the age of 55 years of age is 3.8 times more frequent in individuals having two or more first-degree relatives with high blood pressure (BP) [1], indicating that EH has a strong genetic component.

The prevalence of Hypertension continues to rise despite recent advances in diagnosis and treatment. Approximately 40% of the global adult population aged 25 and above suffer from hypertension [2]. In Sudan, the prevalence of hypertension was reported as 20% in 2011 [3]. Hypertension is a major contributor to the growing global pandemic of CVD and stroke.

Nitric oxide (NO) is important for the anatomical and functional integrity of the vascular endothelium, which is essential for the prevention of atherosclerosis, hypertension, and other CVDs [4]. NO mediates its protective effect on the endothelium by a number of mechanisms including regulation of vasodilatation (either flow-dependent or receptor-mediated vasodilatation), inhibition of leukocyte adhesion to vessels, inhibition of platelet aggregation, and control of muscle cell proliferation [5–7].

NO is produced in endothelial cells by the enzyme, endothelial nitric oxide synthase (eNOS), which catalyzes the conversion of the amino acid arginine to NO and citrulline [8]. Hypertensive patients have reduced levels of NO production, manifested as low levels of urinary and serum nitrate [9].

The gene encoding eNOS, *NOS3*, has been mapped to human chromosome 7q36 and spans approximately 23 kilobases of the genome [10]. *NOS3* represents an interesting candidate gene in relation to EH. The association between *NOS3* and EH has been widely studied, and the disruption of the gene leads to hypertension in mice [11].

Several polymorphic variations of *NOS3* have been identified and investigated. The most clinically relevant *NOS3* variants are rs1799983 (G894 T; Glu298Asp); a variable number tandem repeat (VNTR) in intron 4; and rs2070744 (T-786C) in the promotor region. These variants are associated with CVD, including coronary artery disease [12], myocardial infarction [13, 14], hypertension [15, 16], and stroke [17].

Additional knowledge of *NOS3* gene polymorphisms and their role in hypertension will improve understanding of EH, its common predisposing factors, and potential treatment options. The potential contribution of *NOS3* polymorphisms to the development of hypertension in Sudan has received no attention to date, and no previous studies have addressed this subject. This study aimed to investigate the association of the three polymorphisms in the *NOS3* gene with EH in the Sudanese population.

## Methods

### Patients and control samples

This case control study was conducted in Khartoum, and samples were collected between February 2014 and February 2015. Hypertensive patients (*n* = 260) were enrolled in the study from Samir Health Centre, Soba Teaching Hospital and Fath El Rahman El Bashir Referral Clinics in Khartoum. Patients were selected according to the following criteria: (1) age ≥ 18 years; and (2) established hypertension, defined either by chronic therapy or by blood pressure ≥ 140/90 mmHg according to the National Institute of Health and Care Excellence guidelines [18]. Patients with the following criteria were excluded from the study: (1) any secondary hypertension (excluded by history, clinical examination, creatinine levels in plasma, and urine testing for albuminuria); and (2) evidence of inflammatory processes assessed by the presence of two of the following: tachycardia, hypotension, tachypnea, and high or low temperature [19]. Plasma levels of C-reactive protein were also measured.

Controls (*n* = 144; age ≥ 18 years, blood pressure < 140/90, and without evidence of disease) were recruited mainly from Faculty of Medicine - University of Khartoum and other different institutes in Khartoum and volunteered to participate in the study.

As patients and control groups differed significantly in their age (patients were much older), we omitted all participants with age less than 30 and more than 65 years old. Thereby, 157 patients and 85 controls were included in the statistical calculations.

EH patients and normotensive individuals were consulted about their willingness to participate in the study, and written consent was obtained. Each study participant was interviewed about demographic data, duration of hypertension (for hypertensive patients), family history of hypertension, risk factors for hypertension, medication taken for EH, other medication, smoking, alcohol consumption, and other EH-associated chronic diseases, such as diabetes mellitus, MI, renal diseases, hypercholesterolemia, and stroke, using a structured questionnaire (Additional file 1).

Blood pressure was measured on two occasions in a quiet room after 15 min of resting in a supine position, using a recently calibrated sphygmomanometer.

### DNA sample preparation

Venous blood (5 mL) was drawn from each subject. DNA extraction and genotyping were performed at the Institute for cardiogenetics, University of Lubeck, Germany. DNA extraction was performed using a Qiagen Gentra Puregene Blood Kit (Qiagen, Germany).

### *NOS3* variants and genotyping

rs1799983 (G894 T; Glu298Asp) is a missense variant in exon 7 of *NOS3*. Two alleles of the VNTR in intron 4 have been identified, the larger allele (b) consists of five tandem 27 bp repeats (GAAGTCTAGACCTGCTGCAGGGGTGAG), and the smaller allele (a) consists of four such repeats. Two other rare alleles have been reported, c with six, and d with three, 27 bp repeats. rs2070744 (T-786C) is a polymorphism in the promoter region of *NOS3*. Those three polymorphisms are the best studied in the *NOS3* gene, so they were chosen for genotyping in this study. The literature review done to select these polymorphisms was conducted in 2011. Therefore, recent polymorphisms in *NOS3* associated with EH were not included.

DNA genotyping was performed using TaqMan and polymerase chain reaction restriction fragment length polymorphism (PCR-RFLP) assays. rs1799983 were genotyped using PCR-RFLP as the samples were undetermined with the Taqman method possibly due to multiple variations within the sequence. Two hundred nineteen and 15 samples were genotyped for rs2070744 using Taqman and PCR-RFLP respectively. Genotyping of the VNTR was performed only by PCR-RFLP.

TaqMan assays were performed using a 7900HT Fast Real-Time PCR System (Applied Biosystems, USA), and Sequence Detection System (SDS) software was used to call alleles based on florescence measurements. Genotyping was run in duplicates and the duplicate concordance rate was 100%.

PCR reaction mix (for PCR-RFLP) of 10 μL was prepared. The touch down 61 program was used for the PCR in a Sensoquest thermocycler (Sensoquest GmbH, Germany).

The resulting amplification products were incubated with the restriction enzymes, *Ban*II and *Mbo*I for the rs1799983 SNP, and *Msp*I for the rs2070744 polymorphism. No restriction enzyme was used for the VNTR. Next, the products were mixed with loading dye and SYBR green mix and run in 1.2% (rs1799983 and rs2070744) and 1.8% (VNTR) agarose gels. Estimation of product sizes was carried out with peqGOLD 100 bp DNA ladder (PeqLab, Germany). A Bio-Rad gel documentation system (Bio-Rad Laboratories Inc., USA) was used for gel image capture. The results of the RFLP were determined by two persons reading the gels and in case they disagree about the result, genotyping was repeated. The results of the *Ban*II and *Mbo*I restriction were consistent and concordant.

Primers, restriction enzymes and fragment lengths are presented in Table 1. The primers for rs1799983 and rs2070744 were designed using Primer 3 software [20] and ordered from Eurofins Genomics (Germany). For the VNTR assay (alleles a and b), primers mentioned in the literature were used [21]. For those samples not containing alleles a and b, PCR reactions were cleaned up using NucleoSpin® gel and PCR Clean-up kits (MACHEREY-NAGEL, Germany). Then, amplicons were cloned into the PCR 2.1-TOPO® plasmid vector and recombinants were transformed into competent *Escherichia coli* cells, according to instructions of the TOPO® TA Cloning® kit (Invitrogen, Life Technologies, Germany). Next, PCR was performed using bacterial colonies directly as template. PCR products were visualized by agarose gel electrophoresis, and samples with different lengths were cleaned-up for sanger sequencing (Seqlab company, Germany). The FASTAQ files of the sequencing were aligned with a reference sequence of *NOS3* from Ensemble [22].

**Table 1:** Primers, restriction enzymes, and fragment lengths of the major and minor alleles of rs1799983, rs2070744, and VNTR polymorphisms of *NOS3* gene

	rs1799983		VNTR intron 4	rs2070744
Left primer (5′ to 3′)	AGCCTCGGTGAGATAAAGGA		AGGCCCTATGGTAGTGCCTT	CCCCTGTGGACCAGATGC
Right primer (5′ to 3′)	TCTTGAGAGGCTCAGGGATG		TCTCTTAGTGCTGTGGTCAC	ACATTAGGGTATCCCTTCC
Product size	368 bp			379 bp
Restriction enzyme	*Ban*II	*Mbo*I		*Msp*I
Major allele fragment length (bp)	Cut, two fragments (251 and 117 bp)	Not cut	420 (b)	Two fragments, 146 and 233 bp
Minor allele fragment length (bp)	Not cut, 368 bp	Cut, two fragments (246 and 122 bp)	394 (a)	Three fragments 46, 146, and 187 bp
Heterozygous			394 and 420 bp fragments	Four fragments 46, 146, 187, and 233 bp

### Statistical analysis

Statistical analyses were performed using Statistical Package for the Social Sciences for Windows (SPSS, version 12.0) software. Genotype and allele frequencies were compared between groups by χ^2^ test, and odds ratios (OR) with 95% confidence intervals (CIs) were calculated. Differences in genotype distributions under autosomal dominant and recessive models were also determined using χ^2^ test, and ORs were calculated. Multinomial and binary logistic regressions were performed in genotype comparisons with age, gender, BMI and smoking included as covariates. A χ^2^ test with one degree of freedom was used to determine deviation of genotype distributions from Hardy-Weinberg equilibrium. Values of *P* < 0.05 were considered statistically significant. To eliminate any confounding factors affecting the genetic association study, we also analyzed the distribution of genotypes according to the clinical and demographic characteristics of both groups, including age, gender, BMI, smoking, systolic and diastolic BP, and the presence of complications alongside hypertension (e.g., diabetes, MI, renal failure, hypercholesterolemia, and stroke), using ANOVA for continuous data and χ^2^ or Fisher’s Exact tests for categorical data. Linkage disequilibrium between the three polymorphisms was examined by χ^2^ analysis. The extent of disequilibrium was expressed as *D*′ = *D*/*D*_max_ and Pearson squared correlation coefficient (r^2^).

## Results

### Characteristics of patients and controls

The study included 157 hypertensive patients and 85 controls. The age of hypertensive patients ranged from 32 to 64 years, with a mean of 52.7 years, and that of controls ranged from 31 to 63 years with a mean of 42.1 years. Around 32 % of patients and 81% of controls were male. The systolic BP of patients ranged from 90 to 210 mmHg with a mean (± standard deviation, SD) of 139.8 ± 19.2 mmHg, while that of controls ranged from 90 to 140 mmHg with a mean ± SD of 120.2 ± 10.3 mmHg. The diastolic blood pressure of patients ranged from 60 to 115 with a mean ± SD of 84.6 ± 11.6 while that of controls ranged from 60 to 95 with a mean ± SD of 77.8 ± 8.5. Patients had higher age, BMIs, systolic and diastolic blood pressures, and included a lower percentage of males and smokers than the control group (Table 2).

**Table 2:** Characteristics of patients and controls

	Patients	Controls	*P* value
Mean age ± SD	52.7 ± 7.6	42.1 ± 7.7	< .001
Number of males (percentage)	50 (31.8%)	69 (81.2%)	< .001
Mean systolic BP (mm Hg)	139.8 ± 19.2	120.2 ± 10.3	< .001
Mean diastolic BP (mm Hg)	84.6 ± 11.6	77.8 ± 8.5	< .001
BMI	29.9 ± 6.3	26.5 ± 4.5	0.004
Smoking	6 (3.8%)	23 (30.7%)	< .001
Diabetes	44 (28%)	–	–
Stroke	4 (2.5%)	–	–
MI	6 (3.8%)	–	–
Renal failure	4 (2.5%)	–	–
Hypercholesterolemia	22 (14%)	–	–

*P* values were derived from the χ^2^ test for categorical variables (gender) and student’s t test for continuous variables (age, BMI, and systolic and diastolic blood pressure)

### Genotyping of *NOS3* gene polymorphisms

The distributions of each of the three variants across the study were consistent with Hardy-Weinberg equilibrium (χ^2^ = 0.157, 0.174, and 0.058 for rs1799983, VNTR intron 4, and rs2070744, respectively; all *P* values >0.05).

**Fig. 1:**
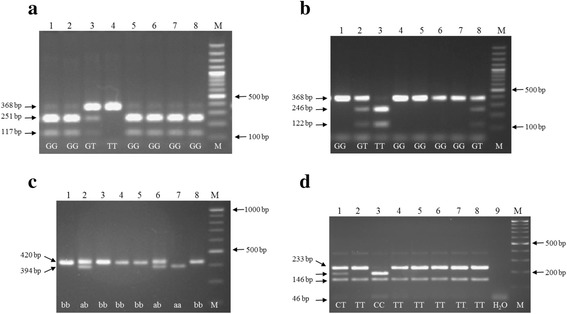
Genotyping of rs1799983 with the restriction enzymes *Ban*II (panel **a**) and *Mbo*I (panel **b**), VNTR intron 4 (panel **c**) and rs2070744 with the restriction enzyme *Msp*I (Panel **d**) using PCR-RFLP

Representative images of PCR-RFLP genotyping results for the rs1799983 polymorphism, which was genotyped for 147 patients and 82 controls (95% of the samples), are presented in Fig. 1 (panel a and b). The GG genotype was more frequent in the control group, while the TT genotype was more frequent in the hypertensive patients; however, the differences between the two groups were not statistically significant (Table 3).

**Table 3:** Genotype distribution of the three *NOS3* polymorphisms among patient and control groups

		Patients, number (%)	Controls, number (%)	
rs1799983	GG	100 (68.0%)	60 (73.2%)	χ^2^ = 0.69 *P* = 0.71
	GT	42 (28.6%)	20 (24.4%)	
	TT	5 (3.4%)	2 (2.4%)	
	Total	147	82	
Intron 4 VNTR	Bb	83 (55%)	50 (64.1%)	χ^2^ = 1.77 *P* = 0.41
	Ab	61 (40.4%)	25 (32.1%)	
	aa	7 (4.6%)	3 (3.8%)	
	Total	151	78	
rs2070744	TT	72 (47.4%)	54 (65.9%)	χ^2^ = 7.74 *P* = 0.02
	TC	70 (46.1%)	23 (28%)	
	CC	10 (6.6%)	5 (6.1%)	
	Total	152	82	

Multinomial logistic regressions were tested for all three polymorphism with age, gender and smoking as covariates and none had an effect on the genotype distribution

The VNTR in intron 4 was genotyped in 154 patients and 83 controls (95% of the samples) (Fig. 1; panel c). Eight samples (three patients and five controls) did not produce results consistent with any of the three genotypes (bb, ab, and aa); therefore, the amplicons from these samples were cloned and sequenced, revealing that they fall into one of two genotypes: ac (one allele with four repeats and the other with six repeats) or bc (one allele with five, and the other with six, repeats). Two patients (1.3%) and one control (1.2%) had the ac genotype while one patient (0.6%) and four controls (4.8%) had the bc genotypes. Consequently three patients (1%) and five controls (3%) had the c allele. Patients and controls with ac and bc genotypes were not statistically analyzed, due to the small numbers involved. Our results indicate that the difference in the distribution of the VNTR genotypes was not statistically significant between the two groups (Table 3).

The *NOS3* rs2070744 polymorphism was genotyped in 152 patients and 82 controls (97% of the samples) (Fig. 1; panel d). The CC genotype was more common among patients than controls (6.6% vs 6.1%), and the TT genotype was more frequent in the control than the patients group (65.9% vs 47.4%), and the differences were statistically significant (*p* = 0.02) (Table 3).

Multinomial logistic regressions for the three polymorphisms showed no effect of either age, gender, BMI or smoking on the distribution of the genotypes. For rs1799983, TT was set as the reference category. Age showed *p* = 0.75 and *p* = 0.38 for GG and GT respectively. Gender had a *p* = 0.79 and *p* = 0.55, BMI: *p* = 0.64 and *p* = 0.48 and smoking: *p* = 0.82 and *p* = 0.66 for GG and GT respectively. Regarding the VNTR polymorphism, aa was set as the reference category. Age showed *p* = 0.94 for bb and *p* = 0.67 for ab. Gender had *p* = 0.84 and *p* = 0.68, BMI, *p* = 0.39 and *p* = 0.29 and smoking *p* = 0.46 and 0.47 for bb and ab respectively. Finally for the rs2070744, CC was set as a reference category and age showed a *p* = 0.68 for TT and *p* = 0.55 for CT. Gender had *p* = 0.68 and *p* = 0.9, BMI *p* = 0.33 and *p* = 0.68 and smoking *p* = 0.57 and *p* = 0.65 for TT and TC respectively.

#### Analysis of associations of *NOS3* polymorphisms with EH under dominant and recessive inheritance models

When dominant and recessive models were assumed for rs1799983, the difference between the EH and control groups was insignificant. The frequency of the T allele was higher in the hypertensive (17.7%) compared with the control group (14.6%), although the difference was not significant with *p* value of 0.40 (Table 4).

**Table 4:** Genotype distribution under dominant and recessive models and allele frequencies of the rs1799983, Intron 4 VNTR and rs2070744 in patients and control groups

			Patients *n*(%)	Controls *n*(%)	
rs1799983	Dominant model	GG	99 (67.3%)	60 (73.2%)	χ^2^ = 0.84 *P* = 0.36 OR (95% CI) = 0.76 (0.42–1.4)
		GT + TT	48 (32.7%)	22 (26.8%)	
	Recessive model	GG + GT	142 (96.6%)	80 (97.6%)	χ^2^ = 0.17 *P* = 0.69 OR (95% CI) = 0.71 (0.14–3.74)
		TT	5 (3.4%)	2 (2.4%)	
	Allele frequency	G	242 (82.3%)	140 (85.4%)	χ^2^ = 0.71 *P* = 0.40 OR (95% CI) = 0.80 (0.47–1.35)
		T	52 (17.7%)	24 (14.6%)	
Intron 4 VNTR	Dominant model	bb	144 (95.4%)	75 (96.2%)	χ^2^ = 0.08 *P* = 0.78 OR (95% CI) = 0.82(0.21–3.3)
		ab + aa	7 (4.6%)	3 (3.8%)	
	Recessive model	bb + ab	83 (55%)	50 (64.1%)	χ^2^ = 1.76 *P* = 0.18 OR (95% CI) = 0.68 (0.39–1.2)
		aa	68 (45%)	28 (35.9%)	
	Allele frequency	b	227 (75.2%)	125 (80.1%)	χ^2^ = 1.42 *P* = 0.23 OR (95% CI) = 1.33 (0.83–2.14)
		a	75 (24.8%)	31 (19.9%)	
rs2070744	Dominant model	TT	72 (47.4%)	54 (65.9%)	χ^2^ = 7.32 *P* = 0.007 OR (95% CI) = 2.14 (1.23–3.74)
		TC + CC	80 (52.6%)	28 (34.1%)	
	Recessive model	TT + TC	142 (93.4%)	78 (95.1%)	χ^2^ = 0.27 *P* = 0.60 OR (95% CI) = 0.73 (0.22–2.4)
		CC	10 (6.6%)	4 (4.9%)	
	Allele frequency	T	214 (70.4%)	131 (79.9%)	χ^2^ = 4.95 *P* = 0.03 OR (95% CI) = 1.84 (1.15–2.93)
		C	90 (29.6%)	33 (20.1%)	

Binary logistic regressions were tested for all the three polymorphisms with age, gender and smoking as covariates and none had an effect on the genotype distribution under dominant and recessive models

There were no significant differences found between the two groups in the distribution of genotypes of the VNTR under dominant and recessive models. The minor allele (a) was more frequent in patients (24.8%) compared with controls (19.9%), although the difference was not statistically significant (Table 4).

When the dominant model was assumed for rs2070744, we found that the TT genotype was more frequent in controls compared with patients, while TC + CC was more frequent in the hypertensive group. The difference was significant with a χ^2^ value of 7.32 and a *P* value of 0.007 and OR (95% CI) of 2.14 (1.23–3.74). There was no significant difference observed under the recessive model. The minor allele (C) was more frequent in patients (29.6%) than controls (20.1%); and the difference was significant (*P* = 0.03, OR (95% CI) = 1.84 (1.15–2.93)) (Table 4).

Binomial logistic regressions for the three polymorphisms showed no effect of age, gender, BMI or smoking status on the distribution of the genotypes considering dominant and recessive models of inheritance. Age, gender, BMI and smoking gave *p* values of 0.52, 0.63, 0.19 and 0.33 respectively in a one-step model for dominant inheritance of rs1799983 and *p* = 0.54, 0.65, 0.42 and 0.46 for recessive inheritance of rs1799983. These variables gave *p* = 0.65, 0.35, 0.66 and 0.39 for dominant inheritance of VNTR and *p* = 0.92, 0.79. 0.29 and 0.19 for recessive inheritance of VNTR. *P* values of 0.91, 0.02, 0.03 and 0.56 were observed for dominant inheritance of rs2070744 and *p* = 0.87, 0.12. 0.46 and 0.26 for recessive inheritance of rs2070744 for the previous variables respectively.

#### Associations between genotypes and general characteristics of the study sample

We studied the associations between the three polymorphisms and the clinical characteristics of the study sample stratified according to age, gender, BMI, systolic and diastolic BP, smoking, and presence of additional complications alongside hypertension (i.e., diabetes mellitus, stroke, MI, renal failure, and hypercholesterolemia). No significant association was found between any of the three polymorphisms with the studied variables.

#### Pairwise linkage disequilibrium between the three polymorphisms

Pairwise linkage disequilibrium between the three polymorphisms was investigated. Allelic association was greatest between rs1799983 and the intron 4 VNTR (D’ = 0.97, r^2^ = 0.06). Weaker associations between rs1799983 and rs2070744 and rs2070744 and VNTR were also detected (D’ = 0.48, r^2^ = 0.12 and D’ = 0.40, r^2^ = 0.02, respectively) (Table 5).

**Table 5:** Pairwise linkage disequilibrium between the markers rs1799983, VNTR, and rs2070744

	D’	r^2^
rs1799983/ VNTR	0.97	0.06
rs1799983/ rs2070744	0.48	0.12
VNTR/ rs2070744	0.40	0.02

D’, standardized measure of disequilibrium; r^2^, Pearson’s correlation coefficient

## Discussion

In this study, three polymorphisms in the *NOS3* gene were genotyped in hypertensive and control groups, to study their association with EH in the Sudanese population.

Regarding rs1799983, the observed minor T allele frequency (MAF) of 0.17 was similar to the global minor allele frequency at this SNP reported by the 1000 genomes project (0.18), although it differed from the reported frequency in the African population (0.07) [23]. Our results were also inconsistent with a previous report by Thomas et al., indicating that the T allele is absent in an African population from Mali [24]. A higher T allele frequency was found in the hypertensive group. However, the difference was not significant. This negative result may indicate a geographic difference within Africa in the distribution of alleles. Many investigations have examined associations between the rs1799983 variant and EH. However, the results have been controversial and inconclusive. Some studies have identified a higher T allele frequency in hypertensive patients and have reported that this allele is associated with resistance to conventional therapy [15, 25]. In contrast, studies in Caucasian populations indicated a higher G allele frequency in the hypertensive group and an association of this allele with the outcome, all-cause mortality [26, 27]. These discrepancies may indicate that either another mutation or SNP is linked to either of the two alleles, or that the observed associations are due to random errors. Conversely, other studies report a lack of evidence for linkage between this polymorphism and EH in the Japanese [28] and Australian [29] populations.

The cause of the association between the rs1799983 variant and CVDs is not well characterized; however, it has been suggested that the resulting replacement of Glu with Asp results in changes to the structure of the eNOS enzyme, increasing its susceptibility to proteolysis [30]. It has also been suggested that this substitution affects the interaction of the enzyme with caveolin-1, thereby affecting its localization and activity [31]. The GG genotype is associated with increased eNOS activity and higher NO levels that are toxic to cells, due to consequent increased superoxide anion production [32, 33].

In the current study, no direct relationship was found between the VNTR and EH. Our results indicate that three hypertensive patients (1%) and five controls (3%) carried the c allele at this locus, which was previously reported by Thomas et al. to be present in Africans and African Americans, but not in Caucasians [24]. In their report, the investigators indicated that this allele is rare; however, our results indicate that it is not rare (>1%) in the Sudanese population [34].

Associations of the a allele of the VNTR in intron 4 of *NOS3* with coronary artery disease and renal disease have been reported [35–37]. However, conflicting data have appeared in the literature concerning the association between this variant and hypertension, even within the same population [15, 38–40]. Moreover, the effect of the a allele on eNOS expression is controversial, with studies reporting both reduced enzyme activity leading to lower NO levels [41] and increased levels of NO production [42] associated with this variant.

The lack of association between either the rs1799983 or VNTR with EH in the Sudanese population could be due either to the small sample size in this study or a genuine indication of a lack of association between these variants and EH in the Sudanese population.

In the present study, the hypertensive group had a significantly higher frequency of CC genotype of the rs2070744 polymorphism compared to the control group (*p* = 0.02). The patients group also had higher frequency of TC + CC genotypes compared with the TT genotype considering a dominant model of inheritance (*P* = 0.04; OR (95% CI) = 0.639 (0.420–0.973)) (Table 4). In addition, the C allele was more frequent in the patients group (*p* = 0.03). There was no association between this genotype and the confounding factors, age, sex, BMI, smoking status, presence of diabetes, MI, stroke, renal failure, or hypercholesterolemia. The observed minor C allele frequency of 0.26 is similar to the global MAF reported by the 1000 genome project (0.23) but different from the MAF for the African population at this SNP (0.14) [23]. The substitution of T to C in this variant at *NOS3* is associated with coronary spasm [43], enhanced coronary vasoconstriction in response to acetylcholine, and impaired endothelium-independent vasodilatation in Caucasians [44]. A study in a Canadian population showed that the CC genotype is associated with higher systolic BP in a healthy cohort and carriers of this allele have a relative risk of developing hypertension of 2.16 (95% CI, 1.3–3.7) compared with non-carriers [45]. In contrast, a study in a Japanese population did not detect any association of this variant with EH [46]. This T to C substitution in the promoter region of *NOS3* reduces its rate of transcription by 50%, both under baseline conditions and in response to hypoxia, and is associated with decreased serum levels of nitrite/nitrate [41, 43]. These effects may be a reflection of the fact that the minor allele can be bound by replication protein A1, which acts as a transcription repressor protein [47], ultimately leading to decreased levels of NO and endothelial dysfunction.

This study has number of limitations that are worth mentioning. The small samples size with potential lack of power to the study is a limitation. Furthermore, cases and controls were significantly different in their age, gender, BMI and smoking status. However, statistical analyses showed that none of these variables had an effect on any of the genotypes distribution.

In this study, we found that all three included *NOS3* polymorphisms are in linkage disequilibrium, and that this effect was strongest between rs1799983 and the VNTR in intron 4, which is expected as they are in close physical proximity to one another. There was also weaker linkage between (rs1799983 and rs2070744), and (rs2070744 and the VNTR). It is unlikely that the VNTR itself has a functional role, as it is in an intronic region. However, it has been suggested to act as a marker for other functional variants elsewhere in the gene. Nakayama et al. first reported that the VNTR is in linkage disequilibrium with rs2070744; hence, the effect of VNTR on *NOS3* mRNA expression, eNOS protein concentration, and enzyme activity is likely to be mediated by the differences in transcriptional efficiency associated with the rs2070744 polymorphism [48–51]. The degree of linkage disequilibrium between the three polymorphisms may help in understanding of the evolutionary divergence of the *NOS3* gene.

## Conclusion

In conclusion, in this study we demonstrate that the rs2070744 polymorphism in the *NOS3* gene promoter is associated with EH in the Sudanese population. We also report the presence of the c allele of the intron 4 VNTR in the Sudanese population and it is not rare. These data indicate that the rs2070744 polymorphism in *NOS3* may be a factor influencing genetic susceptibility to EH in the Sudanese population. Further studies with larger sample sizes and family-based analyses are required to confirm this association.

## Additional file

Additional file 1: Questionnaire data. This excel file contains data collected by interviewed questionnaire from both patients and controls. Also the file contain BMI measurement, blood pressure measurements and lab investigation results. (XLSX 120 kb)

## Abbreviations

BMI: Body mass index
BP: Blood pressure
cGMP: cyclic Guanosine Monophosphate
CI: Confidence interval
CVD: Cardio vascular disease
EH: Essential hypertension
eNOS: Endothelial nitric oxide synthase enzyme
NO: Nitric oxide
*NOS3*: Endothelial nitric oxide synthase gene
OR: Odd ratio
PCR-RFLP: Polymerase chain reaction- restriction fragment length polymorphism
VNTR: Variable number of tandem repeat
